# Male Stress Is Associated with Ovarian and Endometrial Responses in ICSI Cycles: Is Seminal Plasma the Linchpin?

**DOI:** 10.3390/ijms27010534

**Published:** 2026-01-05

**Authors:** Marina Nikolaeva, Alla Arefieva, Alina Babayan, Andrey Romanov, Nataliya Makarova, Liubov Krechetova, Elena Kalinina, Gennady Sukhikh

**Affiliations:** 1 National Medical Research Center for Obstetrics, Gynecology and Perinatology Named After Academician V.I. Kulakov, Ministry of Health of the Russian Federation, 117997 Moscow, Russia; a_arefeva@oparina4.ru (A.A.);; 2 Department of Obstetrics and Gynecology, First Moscow State Medical University Named After I.M. Sechenov (Sechenov University), Ministry of Health of the Russian Federation, 119991 Moscow, Russia

**Keywords:** seminal plasma, male stress, neuroendocrine-immune system, endometrial thickness, ovarian response, IVF/ICSI

## Abstract

Evidence indicates that seminal plasma (SP) has pregnancy-favorable biological effects, but there is no definitive proof that exposure to SP increases pregnancy rates in assisted reproductive techniques. We previously showed that this discrepancy may be due to male stress altering SP composition. This study investigated the association between male stress biomarkers in saliva, serum and SP and key determinants of female fertility in women exposed to their partner’s SP during the intracytoplasmic sperm injection (ICSI) cycle. The prospective pilot study included couples with tubal infertility who had unprotected intercourse during the ICSI cycle, supplemented by intravaginal SP injection on the oocyte retrieval day. Salivary cortisol and seminal noradrenaline were quantified by enzyme-linked immunosorbent assay to assess the activity of the hypothalamic–pituitary–adrenal axis and sympathetic nervous systems. Seminal interleukin-18 was measured using LegendPlex™ technology. Cluster analysis of male stress biomarkers identified two neuroendocrine-immune (NEI) phenotypes, characterized by signs of acute (phenotype-1) and chronic (phenotype-2) stress. Women with NEI phenotype-2 partners had fewer collected, mature, and fertilized oocytes, thinner endometrium, and significantly lower pregnancy rates (18.2%) compared to those with NEI phenotype-1 partners (84.6%). These data may suggest a dual role for SP in female fertility, depending on the type of male stress.

## 1. Introduction

A recent epidemiological study estimates the global prevalence of infertility among couples of reproductive age to be approximately 17.5% [1].

Artificial reproductive technologies (ARTs), including In Vitro Fertilization (IVF) and Intracytoplasmic Sperm Injection (ICSI), are primary approaches to addressing infertility. ICSI was initially developed primarily for severe cases of male factor infertility, such as oligoasthenoteratozoospermia or non-obstructive azoospermia. However, it is now widely used even in non-male factor cases, comprising 75–80% of IVF/ICSI procedures among reporting clinics in the ESHRE European IVF Monitoring data [2]. Despite advancements in the embryonic stage of these programs that enable the selection of high-quality embryos, pregnancy rates following embryo transfer remain relatively low. According to the European IVF Monitoring Consortium in 2020, the pregnancy rate per transfer was 33.2% for IVF and 33.0% for ICSI [2]. This limited effectiveness of ART is likely attributable to losses occurring during embryo implantation, largely due to impaired endometrial receptivity, which depends on the balanced regulation of endocrine and immune responses [3,4,5,6]. Consequently, developing strategies to overcome this “last barrier” to IVF success [7] has become a major focus of reproductive research.

Couples undergoing IVF/ICSI treatment are advised to use condoms during sexual intercourse or to abstain from intercourse throughout the entire cycle, preventing contact of the female reproductive tract (FRT) with both spermatozoa and seminal plasma (SP). However, recent studies have significantly advanced the understanding of the critical role of SP in fertility. Key components of SP include fructose, citric acid, zinc, enzymes, prostaglandins, amino acids, potassium ions, and diverse signaling proteins with immunological activities. Animal studies have demonstrated that SP substantially enhances endometrial function and improves embryo implantation rates [8]. In humans, SP has been shown to activate multiple transcriptional regulatory pathways that induce morphological and functional changes in endometrial cells, playing a crucial role in successful embryo implantation [9,10,11,12,13,14,15,16,17,18]. Therefore, it is reasonable to suggest that the lack of FRT exposure to SP during the IVF/ICSI cycle may contribute to the reduced effectiveness of these treatments.

Although many research groups have investigated SP application in ART, evidence remains contradictory [19,20].

Previously, we showed that this discrepancy may primarily result from differences in the content of stress biomarkers in SP derived from fertile and/or healthy donors used in experimental studies, compared to SP from IVF/ICSI-treated patients participating in clinical trials [21]. Moreover, the association between male stress, SP composition, and ART outcomes was established in couples who had sexual relations during the IVF/ICSI cycle, which was supplemented by intravaginal intromission of SP on the day of ovum pick-up (Day-OPU) [21].

Stress is broadly defined as the body’s response to any physical or psychological stimulus (stressor) that disrupts homeostasis [22], mediated by the activation and complex interactions of the sympathetic nervous system (SNS), the hypothalamic–pituitary–adrenal (HPA) axis, and the immune system [23,24]. Additionally, the SNS consists of the sympathetic adrenomedullary and sympathetic neural axes, both of which are activated immediately following the onset of stress. Stimulation of the SNS leads to the release of adrenaline (ADR) primarily from the adrenal medulla and noradrenaline (NA) mainly from the sympathetic nerve terminals and nonsynaptic varicosities into the extracellular space [25]. The glucocorticoid hormone cortisol (CORT) is secreted by the adrenal cortex and serves as a principal output of the HPA axis. Salivary CORT levels have been demonstrated to be a reliable indicator of HPA axis function [26]. Stress induces proinflammatory cytokine production through neuroendocrine pathways involving the HPA axis and SNS activity [27,28,29,30]. Interleukin-18 (IL-18) is increasingly recognized as a marker of inflammatory reactions associated with the stress response [31,32].

The HPA axis, SNS and the immune system are closely interconnected, forming an integrated network that coordinates the body’s response to stress and maintains homeostasis [33,34,35,36,37]. This multicomponent dynamic regulatory network is recently termed the neuroendocrine-immune (NEI) system [38]. Balanced interactions among stress-related systems maintain homeostasis during short-term acute stress by rapidly mobilizing adaptive responses [33,39]. Disruptions in this network during prolonged chronic stress are associated with an increased risk of physical and mental illnesses [39,40,41,42,43]. In this study, we aimed to evaluate the validity of the integrated multi-system stress response model specifically in the context of reproductive health.

Numerous studies have suggested that male infertile patients may experience stress related to infertility and the IVF/ICSI treatment [44,45,46,47,48]. The contribution of male stress to pregnancy failure is largely explained by the deterioration in sperm quality, which manifests as decreased semen volume, lower sperm count, reduced sperm motility, and increased sperm DNA damage [49,50,51,52,53]. However, several recent large-scale studies have not found conclusive evidence that male psychological stress is associated with reduced testicular function and erectile dysfunction [54] or IVF/ICSI outcomes [55].

We have previously demonstrated that the stress experienced by male IVF/ICSI patients is reflected by stress biomarkers measured not only in serum and saliva but also in SP [21]. Moreover, levels of key biomarkers of stress—salivary CORT, seminal NA, and the proinflammatory cytokine IL-18—were identified as strong prognostic factors predicting IVF/ICSI success in women exposed to their partner’s SP during an ICSI cycle [21,56]. SP can therefore be considered a unique bodily fluid that acts both as a sensor of the male stress experienced by IVF/ICSI patients before and during semen donation and as a transmitter of NEI signatures of stress to the FRT.

Evidence suggests that a counter-current exchange mechanism may facilitate the rapid transport of SP components into the FRT, enabling seminal molecules to reach the uterus [57,58]. Effects of SP on the endometrium have been observed in both animal [59,60,61] and human [9,10,11,12,13,14,15,16,17,18,62,63] studies. In animals, ovarian function has also been shown to be influenced by seminal components [64,65]. We propose that stress biomarkers in SP, transmitted through sexual intercourse during the proliferative phase of the endometrium in an IVF/ICSI cycle, may influence reproductive outcomes, affecting ovarian and endometrial responses to stimulation.

The human endometrium is a highly dynamic tissue that undergoes cyclic remodeling during the proliferative phase of both the menstrual and IVF/ICSI cycles, closely linked to ovarian function [66,67,68]. Endometrial cell proliferation and angiogenesis, regulated by ovarian steroid hormones, contribute to endometrial thickening, thereby creating an optimal environment for embryo implantation [69]. The number of collected, mature, and fertilized oocytes, as well as endometrial thickness (EMT), are key female reproductive determinants related to IVF/ICSI success in standard ART protocols [70,71].

Thus, we hypothesize that the effect of male stress on IVF/ICSI outcomes is mediated both by the direct impact of seminal stress biomarkers on the endometrium and by the modulation of ovarian stimulation, leading to altered ovarian signaling and consequently affecting endometrial growth. The aim of this study was to investigate the relationships between key stress biomarkers in male ICSI patients and the ovarian and endometrial responses to stimulation in women exposed to their partner’s SP during ICSI cycles.

## 2. Results

### 2.1. Demographic, Clinical, and Reproductive Characteristics Related to ICSI Outcomes

Of the 24 female patients exposed to SP during ICSI cycles, 13 (54.2%) achieved clinical pregnancy. There were no significant differences in the demographic and clinical profiles of patients related to ICSI outcomes (Table 1).

Differences in the follicle-stimulating hormone (FSH) to luteinizing hormone (LH) ratio approached statistical significance (Table 1).

There were no significant differences in conventional semen parameters between patients with successful and unsuccessful ICSI outcomes. (Table 1).

The comparison of stress biomarkers between male partners of women who achieved pregnancy and those who did not is shown in Table 1. Significant differences were observed in salivary and serum CORT, with higher levels in patients from the successful ICSI group. No significant intergroup differences were found in the concentration or total content of seminal CORT. Differences in both the concentration and total content of NA in SP approached statistical significance. Levels of other seminal catecholamines, adrenaline (ADR) and dopamine (DOP), did not differ significantly between groups. Additionally, no significant intergroup differences were observed in the concentration or total content of seminal IL-18. No significant differences were found in EMT and the number of fertilized oocytes, while differences in the number of collected and mature oocytes were near statistical significance (Table 1).

### 2.2. Neuroendocrine-Immune Phenotypes in Male ICSI Patients

**Figure 1:**
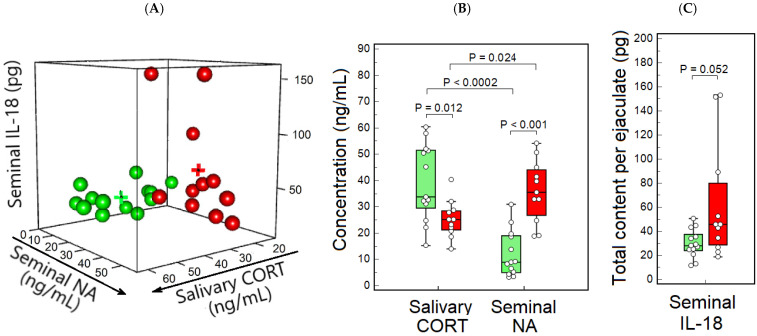
Cluster analysis of the biomarkers representing hypothalamic–pituitary–adrenal axis activity (measured by salivary CORT), sympathetic neural activity (measured by seminal NA), and immune response (measured by seminal IL-18) in male ICSI patients. (**A**) Cluster 1 (*n* = 13, green) and Cluster 2 (*n* = 11, red) are shown in corresponding colors, with centroids marked by crosses; (**B**) Significant intercluster differences in concentration of salivary CORT and seminal NA; (**C**) The near-significant intercluster difference in total seminal IL-18 content per ejaculate; (**B**,**C**) Results are depicted as box-and-whisker plots in green and red, representing parameters within Cluster 1 and Cluster 2, respectively. CORT: cortisol; NA: noradrenaline; IL-18: interleukin-18.

We evaluated the integrated profile of NEI system activity by measuring salivary CORT, seminal NA, and total IL-18 content per ejaculate, which are key biomarkers of the HPA axis, SNS, and immune system status, respectively. These biomarkers have been previously shown to be prognostically significant indicators of pregnancy outcomes in IVF/ICSI programs [21,56]. Cluster analysis of stress biomarkers identified two distinct groups within the male ICSI patient population (Figure 1A). The centroids of Clusters 1 and 2 corresponded to salivary CORT levels of 39.2 ng/mL and 25.3 ng/mL, seminal NA levels of 12.1 ng/mL and 35.9 ng/mL, and total IL-18 content in semen of 30.0 pg and 62.2 pg, respectively. The proportions of male ICSI patients in Clusters 1 and 2 were comparable (54.2% vs. 45.8%, respectively; *p* > 0.05), with their female partners assigned to the corresponding clusters.

The results of the analysis of stress biomarkers in the two clusters are presented in Figure 1B,C and Table 2. Salivary CORT levels in Cluster 1 were significantly higher compared to Cluster 2 (Figure 1B, Table 2). No significant differences between clusters were observed in the concentration of serum CORT nor in the concentration or total content of seminal CORT (Table 2). Seminal NA concentration and total NA content were significantly lower in Cluster 1 relative to Cluster 2 (Figure 1B, Table 2). Thus, in Cluster 1, salivary CORT levels were much higher than seminal NA levels, whereas in Cluster 2, salivary CORT levels were lower than seminal NA levels (Figure 1B). Although seminal IL-18 levels were lower in Cluster 1 relative to Cluster 2, the difference approached statistical significance (Table 2, Figure 1C).

It should be noted that, in patients from Cluster 1, salivary CORT levels were 3.8 times higher than seminal NA levels, whereas in those from Cluster 2, salivary CORT levels were 1.4 times lower than seminal NA levels (Figure 1B). In Cluster 1, seminal CORT concentrations were 5.9 times higher than seminal NA concentrations (Table 2, *p* = 0.0002). No significant differences were detected in these parameters in Cluster 2 (Table 2, *p* > 0.05).

So, cluster analysis of stress biomarkers in male ICSI patients has revealed two distinct NEI phenotypes. Males with NEI phenotype-1 exhibited higher HPA axis activity before semen collection, as indicated by elevated salivary CORT levels, but lower sympathetic activity at ejaculation, as indicated by reduced seminal NA levels, compared to those with NEI phenotype-2.

### 2.3. Demographic, Clinical, and Reproductive Characteristics of ICSI Couples by Male NEI Phenotype

Cluster analysis of stress biomarkers related to the activity of the SNS, HPA axis, and immune system identified two distinct NEI phenotypes in male ICSI partners.

**Figure 2:**
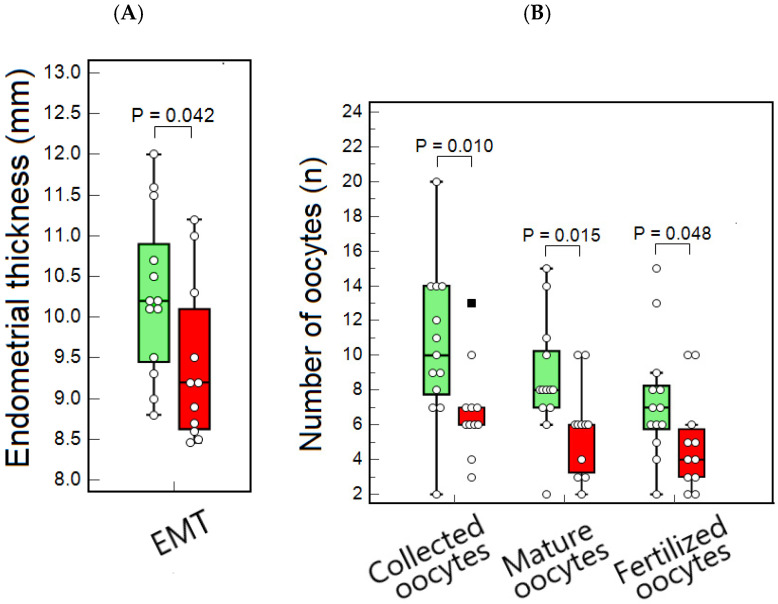
Reproductive characteristics of female partners of male ICSI patients with different neuroendocrine-immune (NEI) phenotypes. (**A**) Significant intergroup differences in EMT; (**B**) Significant intergroup differences in the number of collected, mature, and fertilized oocytes. Results are depicted as box-and-whisker plots in green and red, representing parameters of female ICSI patients assigned to the male NEI phenotype-1 and NEI phenotype-2 groups, respectively.

We compared women and their male partners across these NEI phenotypes regarding demographic, clinical, and reproductive characteristics (Table 3, Figure 2).

Both female and male ages were significantly lower in Cluster 1 compared to Cluster 2. Furthermore, the duration of infertility was shorter among patients within Cluster 1. LH was significantly higher in Cluster 1, while the FSH/LH ratio was significantly lower in Cluster 2. No significant differences were observed in FSH and AMH (anti-Müllerian hormone) levels between clusters. EMT in women related to Cluster 1 was higher compared to that in Cluster 2 (Table 3, Figure 2A). The numbers of total, mature, and fertilized oocytes retrieved from women in Cluster 1 were significantly higher compared to those in Cluster 2 (Table 3, Figure 2B). ICSI outcomes in women were strongly and significantly different between clusters (Table 2). Pregnancy rates were markedly higher in Cluster 1 than in Cluster 2, with rates of 84.6% (11/13) and 18.2% (2/11), respectively.

### 2.4. Univariate Correlation Analysis Between Stress Biomarkers and Demographic, Clinical, and Reproductive Characteristics of ICSI Patients

Correlations between key male stress biomarkers and patients’ demographic and clinical characteristics are summarized in Table 4. Salivary CORT was negatively correlated with male age and showed a trend toward statistical significance with female age and duration of infertility.

In contrast, NA concentration in SP positively correlated with female age, male age, the duration of infertility and the FSH/LH ratio. It also showed a trend toward significance with LH levels. Additionally, seminal NA showed a negative correlation with EMT, which was very close to statistical significance, a strong negative correlation with the total number of collected and mature oocytes, and a weak but significant correlation with fertilized oocytes.

Seminal IL-18 was also positively correlated with male age and showed a trend toward statistical significance with female age.

### 2.5. Correlations Between Demographic, Clinical, and Reproductive Characteristics of ICSI Patients

**Figure 3:**
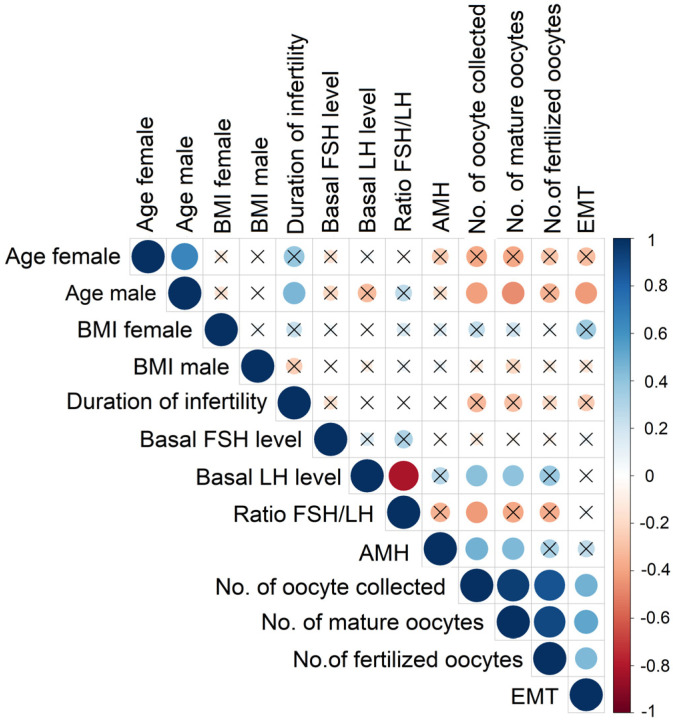
Visualization of the correlation matrix for demographic, clinical, and reproductive characteristics of male and female ICSI patients. Red indicates positive correlation, and blue reflects negative correlation. The size of the circle reflects the value of Spearman’s rank correlation coefficient, and boxes without an “×” are significant (*p* < 0.05). BMI: body mass index; FSH: follicle-stimulating hormone; LH: luteinizing hormone; AMH: anti-Müllerian hormone; EMT: endometrial thickness.

Correlations between demographic and clinical characteristics of both male and female participants are shown in Figure 3. A strong positive correlation was found between male and female age. Duration of infertility in couples also positively correlated with male age and showed a trend toward statistical significance with female age.

Male age showed negative correlations with EMT as well as the numbers of collected and mature oocytes. Correlations between female age and the numbers of collected and mature oocytes approached statistical significance.

Basal LH levels positively correlated with the number of collected and mature oocytes. The FSH/LH ratio positively correlated with AMH levels and with the numbers of collected, mature, and fertilized oocytes. AMH levels also showed positive correlations with the numbers of collected and mature oocytes.

Significant positive correlations were found among the numbers of collected, mature, and fertilized oocytes, all of which correlated positively with EMT.

## 3. Discussion

To our knowledge, this is the first demonstration that the male NEI network is strongly associated with key female reproductive determinants influencing ICSI success in women exposed to their partner’s SP during the ICSI cycle.

It is well established that basal LH levels, the FSH/LH ratio, EMT, and the number of collected, mature, and fertilized oocytes are associated with IVF/ICSI success in standard treatment protocols without additional experimental interventions [70,71]. In our ICSI protocol incorporating contact with SP, we found a near statistically significant association between pregnancy achievement and the number of mature and fertilized oocytes, as well as the FSH/LH ratio. Notably, male stress patterns emerged as the strongest predictor of ICSI success, showing strong associations with the number of collected, mature, and fertilized oocytes, EMT, basal LH levels, and the FSH/LH ratio.

In the present study, cluster analysis of stress biomarkers reflecting HPA axis, SNS, and immune system activity identified two distinct NEI phenotypes among male ICSI partners, characterized by differing balances of salivary CORT, seminal NA, and IL-18. Male ICSI patients with low salivary CORT exhibited significantly higher seminal NA and IL-18 levels than those with high salivary CORT.

Previously, distinct patterns of stress-response system activity have been identified in subgroups of animals [72] and humans [73], recently termed immune-neuroendocrine phenotypes [74], neurohormonal immune phenotypes [73], or NEI phenotypes [38]. These phenotypes represent distinct stress biomarker profiles that are consistent within subgroups but differ significantly between subgroups within the same human or animal population.

The concept of stress-induced NEI phenotypes refers to how organisms adapt to environmental stressors by altering physiological and behavioral responses through an integrated NEI pathway [38]. Our data suggest that two male NEI phenotypes may be linked to reproductive programming in women exposed to SP during ICSI. A significantly lower number of collected, mature, and fertilized oocytes, along with decreased EMT, were observed in women associated with NEI phenotype-2 compared to NEI phenotype-1, with pregnancy rates differing significantly between the groups (18.2% vs. 84.6%, respectively).

The lack of observed effects of male stress on IVF outcomes, as suggested by a previous study [55], may be attributed to the use of standard protocols that exclude contact between SP and the FRT during the IVF/ICSI cycle. In this study, as well as in our previous works [21,56], we confirmed a strong correlation between male stress signatures and IVF/ICSI outcomes using treatment protocols that included contact with SP. These data support the hypothesis that the FRT can receive and respond to seminal stress-related signaling molecules, potentially influencing IVF/ICSI outcomes.

It was shown that the concentration of seminal NA in male patients with NEI phenotype-2 is nearly four times higher than in those with NEI phenotype-1. Additionally, we found a negative correlation between seminal NA levels and the number of collected, mature, and fertilized oocytes. These results suggest that seminal NA may be a key factor influencing the effect of SP on ICSI outcomes, while oocyte numbers remain crucial for IVF success [75,76].

Seminal NA can mimic the effects of endogenous NA, a key neurotransmitter involved in the sympathetic regulation of ovarian function through its release from sympathetic nerves innervating the ovary [77]. The effects of endogenous NA on the ovaries depend on its concentration. At moderate levels, NA supports ovarian function by activating β2 adrenoreceptors (ARs) on granulosa cells, thereby regulating steroid hormone production and follicle development [77,78,79,80]. However, high concentrations of NA impair ovarian function. Follicular NA levels negatively correlate with the presence of good-quality embryos and IVF outcomes [81]. Elevated NA contributes to the development of polycystic ovary syndrome (PCOS) [82,83]. Recent experimental studies highlight an evolutionarily conserved role of noradrenergic signaling in maintaining oocyte quiescence under stress conditions [84].

It is well established that folliculogenesis is regulated both by local ovarian factors and systemic signals from the hypothalamic-pituitary-gonadal (HPG) axis. Neuronal input controls the pituitary secretion of gonadotropins—FSH and LH—which drive follicle growth. A high basal FSH/LH ratio is predictive of a poorer ovarian response to controlled ovarian stimulation and lower pregnancy rates in women undergoing a gonadotropin-releasing hormone (GnRH) antagonist protocol [85]. In this study, using the same protocol, female partners of men with NEI phenotype-2 exhibited lower basal LH levels, a higher FSH/LH ratio, and fewer oocytes retrieved. A strong positive correlation was observed between seminal NA levels and the FSH/LH ratio, with seminal NA also showing a near-significant correlation with LH. These data suggest that SP may influence ovarian function through pathways involving the central nervous system.

Previous animal studies support this hypothesis, demonstrating that seminal factors quickly enter the female bloodstream after mating and influence folliculogenesis. This effect may occur by stimulating GnRH secretion in the hypothalamus or directly inducing LH release from the pituitary gland [86,87]. Additionally, experimental evidence indicates that exogenous NA dose-dependently inhibits the effect of GnRH on LH secretion [88,89]. Our findings align with these observations, suggesting that excess seminal NA in the female bloodstream may disrupt the HPG axis, thereby reducing LH secretion and ovarian response during IVF/ICSI.

In natural cycles, follicular waves synchronize endometrial growth through estrogen signaling [66], but ovarian stimulation during IVF/ICSI cycles alters this dynamic. Stimulation protocols can affect the relationship between EMT and follicular development, with variations depending on the medications used and their methods of administration [90]. The present data indicate a significant positive correlation between EMT and the number of collected, mature, and fertilized oocytes, suggesting that stress-related components of SP, when introduced into the FRT, may orchestrate these key reproductive determinants.

It was shown that EMT in female partners of men with NEI phenotype-2 is lower than in those with NEI phenotype-1. Additionally, a near-significant negative correlation was found between seminal NA and EMT. While a strong negative correlation was observed between NA and the number of oocytes retrieved, the effect of NA on EMT may be mediated through its impact on folliculogenesis.

In addition, seminal NA may affect the endometrium by mimicking the direct effects of endogenous NA, which plays crucial roles in decidualization, implantation, and immune regulation [91,92,93]. It is well established that the expression of ARs and their activation by NA are closely related to endometrial function [93,94,95], whereas overexpression of NA plays a critical role in impaired decidualization [95,96].

Along with the effects of SP on folliculogenesis and endometrial growth, decidualization—which coincides with the development of an optimally receptive endometrium—may also be influenced by seminal factors. Long-term effects of SP treatment on in vitro decidualization have been observed up to 12 days post-exposure and confirmed in experimental human and animal studies [10,11]. Therefore, the transfer of stress-related seminal factors into the FRT during intercourse in the proliferative phase, as well as artificial SP application on Day-OPU, may affect decidualization—a process that typically begins about 3–5 days after ovum pick-up and is crucial for IVF/ICSI success.

It is highly probable that the differences observed in seminal NA concentrations between the two phenotypes are attributable to HPA axis activity prior to ejaculation. Cortisol-mediated inhibition of sympathetic neural activity is considered a key mechanism in the adaptation to acute stress [97,98,99]. Moreover, existing evidence indicates that anticipatory stress, manifested by an increase in CORT, is considered an important physiological strategy that prepares humans for psychological and physical demands by conferring protection against subsequent stressors [100,101,102]. Conversely, chronic stress accompanied by a blunted CORT response leads to sympathetic overactivation [97,99,103]. These neuroendocrine mechanisms appear relevant to male reproductive function.

Previously, we showed that the anticipatory increase in CORT likely occurs before masturbation in fertile donors and in males from the ICSI successful group [21]. In male patients classified as NEI phenotype-1, salivary CORT levels before masturbation were significantly higher than seminal NA levels. In contrast, patients with NEI phenotype-2 exhibited an inverse relationship between these parameters. These results suggest that activation of the HPA axis before semen collection may counterbalance excessive sympathetic nervous system reactions occurring at ejaculation.

The peak sympathetic neural response integrates into the complex neuroendocrine reactions that induce ejaculation [104,105,106], playing a crucial role in normal male sexual function. The strong sympathetic reaction during orgasm and ejaculation in healthy men, described as a generalized “sympathetic storm” [107], is evidenced by increased circulating NA levels [104,105], pronounced elevations in heart rate and blood pressure [104,108], and extremely high concentrations of NA in SP [21,109]. It has been established that abnormal hyperactivation of orgasmic sympathetic tone may adversely affect both sexual [110] and cardiovascular [111] functions.

Thus, an appropriate CORT level may prevent excessive SNS reactions in NEI phenotype-1 and maintain a pregnancy-favorable concentration of NA in SP. In contrast, the blunted HPA axis response attributed to NEI phenotype-2 results in SNS overactivation, associated with an abnormal SP composition.

The SNS, as well as the HPA axis, orchestrates immune responses throughout the body. It is well established that locally released NA can evoke an inflammatory response via activation of ARs [25,112]. On the contrary, CORT has potent anti-inflammatory and immunosuppressive properties [113,114]. Consequently, signs of inflammatory reactions, including elevated cytokine production, are associated with sympathetic overactivation and hypoactivity of the HPA axis during chronic stress and stress-related disorders [97,115,116,117,118,119].

These patterns of chronic stress appear to be characteristic of men related to NEI phenotype-2, exhibiting HPA axis hypoactivity, overactivation of the SNS, and peripheral inflammatory response, as indicated by blunted salivary CORT, heightened seminal NA, and slightly elevated seminal IL-18 levels, respectively.

SP contains a broad array of immunologic factors, including cytokines, chemokines, and growth factors [120]. Although some seminal cytokines and chemokines are considered pregnancy-favorable in both humans and animals [8,11,63,121,122], overexpression of proinflammatory cytokines in SP can adversely affect female reproductive function [123,124,125]. Our previous research revealed a negative association between the total content of IL-18 in SP and IVF/ICSI outcomes [56], as well as with EMT [126]. Endometrial growth is closely associated with immune cell migration, and immune-related molecular changes actively contribute to tissue remodeling and angiogenesis [127,128]. In line with these data, our previous research demonstrates that seminal IL-18 may be associated with the migratory activity of circulating regulatory T cells [129].

Recent studies support the dual role of endogenous IL-18 in endometrial function [130]. Endogenous IL-18, which exerts concentration-dependent effects on the endometrium, acts as a local immune modulator crucial for normal endometrial function [131,132] and is implicated in pathologies such as adenomyosis [133], endometriosis [134], and PCOS [135]. Thus, combined with endogenous IL-18, seminal IL-18 may play a significant role in shaping the immune microenvironment and endometrial growth in a concentration-dependent manner.

Previous studies have revealed complex interactions between the HPA and HPG axes, including inhibition of gonadal androgen production by CORT [136,137]. Thus, blunted HPA activity in NEI phenotype-2 may be related to androgen overexpression. Androgens exhibit antiproliferative effects on endometrial cells [138]. Therefore, in men with NEI phenotype-2, excess seminal androgens entering the FRT during the follicular phase, along with NA and IL-18, may inhibit regenerative processes and consequently reduce EMT.

In line with these proposals, our previous research established a positive association between IL-18 and androgen levels in the SP of male ICSI patients [139], as well as a negative association between these immunohormonal markers and EMT in women exposed to their partner’s SP during the ICSI cycle [126].

The findings indicate that in male ICSI patients, the integrated activity of stress-related systems and the HPG axes modulates the composition of SP. This modulation correlates with key reproductive parameters such as folliculogenesis, EMT, and ultimately, ICSI outcomes in their female partners.

The identification of two stress-related NEI phenotypes in males undergoing ICSI—one resembling an acute adaptive stress response and the other reflecting an exhaustion stage of chronic stress—supports the concept that acute and chronic stress exert divergent facilitatory and inhibitory effects, respectively, on both male and female reproductive functions [140,141,142,143,144,145].

Recent findings indicate that men undergoing IVF/ICSI treatment experience mild to moderate anxiety and perceived stress before sperm collection [146]. Acute adaptive stress before masturbation in IVF/ICSI patients may be triggered by environmental factors, such as the hospital atmosphere, interactions with staff and patients, the location of the collection room, and noise [147]. Additionally, venous blood sampling or the anticipation of venipuncture can increase HPA axis activity and act as a potential stressor [148]. Moreover, it has been confirmed that stress associated with the perceived importance of producing a semen specimen increases significantly in male IVF patients on the Day-OPU compared to the pre-IVF sampling period [147]. Based on these data, the findings presented in this article suggest that acute stress in male patients with NEI phenotype-1 may have a beneficial effect on folliculogenesis and endometrial development, mediated by SP transmitting the signatures of acute stress to the FRT. The beneficial effect of precoital acute stress for reproductive success was confirmed in animal studies [149,150]. Recent data also indicated that acute stress may improve semen parameters in humans [142,144].

Chronic stress is attributed to prolonged periods of continuous stress and/or the cumulative number of stressful life events. Couples exhibiting NEI phenotype-2 had a longer duration of infertility compared to those with NEI phenotype-1. Therefore, chronic stress in male patients with NEI phenotype-2 likely results from an extended period of infertility and/or infertility treatment. This study supports an association between chronic stress and aging in ICSI patients by demonstrating a positive correlation between male age and duration of infertility, along with a negative correlation between male age and salivary CORT. Natural aging alone does not significantly alter HPA axis activity and may even be linked to increased CORT reactivity to stress [151,152], whereas CORT negatively correlates with the number of stressful life events [153]. These findings suggest that, within the context of the ICSI protocol used in this study, male age should be considered a confounder rather than a causal factor affecting female reproductive function. It should be noted that most studies using standard protocols that exclude contact with SP have also not found male age to affect ICSI outcomes [154,155]. Hence, it is highly likely that the negative correlations observed between male age and total, mature, and fertilized oocyte counts, as well as EMT, do not reflect causative relationships.

Although women associated with different phenotypes had statistically different average ages of 30.0 and 32.0 years, no significant correlation was found between age and key female reproductive parameters. This aligns with data showing that female age under 35 only modestly correlates with some reproductive characteristics, with significant declines typically occurring at age 35 or older [156].

Experimental studies have demonstrated a clear role of paternal chronic stress in programming stress-related behavioral and cognitive abnormalities in offspring through epigenetic changes in spermatozoa, which are transmitted to the oocyte [157,158,159]. Therefore, it cannot be excluded that the transfer of seminal biomarkers of male chronic stress into the female reproductive system may disrupt embryo implantation, potentially preventing the transmission of harmful epigenetic changes that might otherwise compromise future generations.

**Figure 4:**
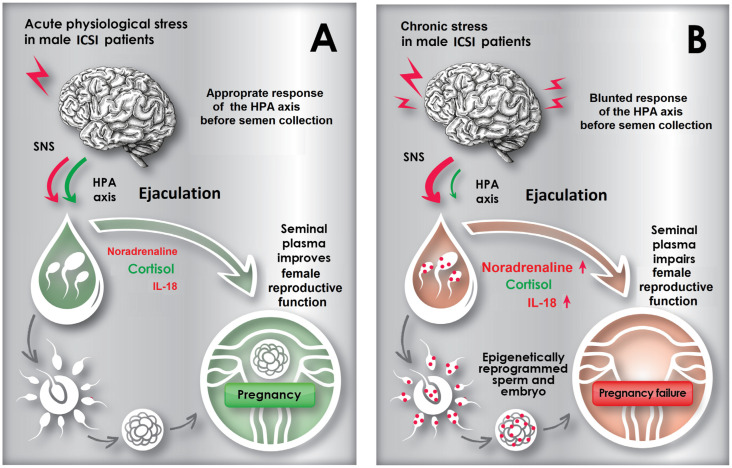
A putative model of the relationships between male stress in ICSI patients, SP composition, and pregnancy establishment in women exposed to their partner’s SP during the ICSI cycle. (**A**) In male ICSI patients experiencing acute stress before semen collection, elevated HPA axis activity—manifested by high salivary CORT prior to masturbation and persisting throughout the sexual cycle—counterbalances sympatoneural activity, which triggers orgasm and ejaculation. This balanced interplay of stress systems, detectable by the biomarker pattern and designated as NEI phenotype-1, maintains optimal levels of seminal NA, CORT, and IL-18. Stable biomarker profiles in SP, whether from masturbation or intercourse during the proliferative phase, stimulate folliculogenesis, endometrial growth, and endometrial receptivity. (**B**) In male ICSI patients experiencing chronic stress before semen collection, ejaculation occurs against a background of HPA axis hypoactivity, manifested by sympathoneural hyperactivity at ejaculation and NA overexpression in SP. This discoordination between the HPA and sympathetic neural axes, characteristic of NEI phenotype-2, is accompanied by signs of inflammation and elevated proinflammatory IL-18 in SP. The imbalance of stress biomarkers in SP links paternal chronic stress to reduced folliculogenesis, endometrial growth, and receptivity, preventing implantation of an epigenetically altered embryo that has received epigenetic signatures of chronic stress from spermatozoa. ICSI: intracytoplasmic sperm injection; HPA: hypothalamic–pituitary–adrenal; SNS: sympathetic nervous system; IL-18: interleukin-18.

Thus, male stress may significantly influence female reproductive function through complex mechanisms, including the effects of SP on folliculogenesis and endometrial growth. To illustrate this hypothesis, the discussion includes a schematic figure (Figure 4).

The obtained data highlight the potential role of male stress in human reproduction. However, these findings do not establish a causal relationship between male stress-related factors and folliculogenesis or EMT, as female stress alone can independently affect ovarian function. The concept of a “stressed couple” [160] is supported by observed associations between partners’ emotional responses during IVF cycles [44,46] and correlations in stress biomarker levels within couples [161]. Thus, SNS and HPA axis activity in male partners may reflect the activity of the female stress response system, potentially interfering with folliculogenesis by suppressing LH secretion through distinct neural and endocrine pathways [162]. Therefore, the influence of stress from both partners on folliculogenesis and EMT cannot be excluded. It must be highlighted that despite the large number of publications addressing the effects of female stress on the effectiveness of ART in humans, the data remain highly contradictory. Some studies have revealed an association between female stress and IVF outcomes [71,163,164]. However, other studies suggest that although stress often increases in females during IVF cycles, there is no clear, direct association between early-cycle stress and IVF outcomes [165,166,167]. It should be noted that in women, estradiol has been proposed to exert a potential stress-reducing effect [168], with its levels directly proportional to the number of developing follicles and eggs produced [169].

The relationship between key female determinants of ICSI success and semen composition, assessed by a single-point sperm analysis on the Day-OPU, was interpreted in the context of SP effects on the FRT during sexual intercourse in the proliferative phase of the ICSI cycle. These observed associations likely reflect low within-subject variability in NEI system activity patterns, leading to stable expression profiles of stress biomarkers in SP produced during both masturbation and sexual intercourse.

A strength of this study is the use of an ICSI protocol supplemented by intercourse during the ICSI cycle and intravaginal seminal plasma application on oocyte retrieval day, enabling assessment of cumulative SP exposure effects similar to those in natural fertilization cycles.

Another major advantage of this research is the use of a multisystemic approach to evaluate the integrated activity of the general NEI regulatory system. Male psychological stress and its association with ART outcomes are primarily assessed using questionnaires [44,45,46,47,48,49,55,170], with only a limited number of studies measuring the activity of individual stress-related systems [48,171,172]. Therefore, our use of objective measures reflecting the activity of major, interconnected stress response systems represents a key strength of this study. Objective parameters are crucial for assessing stress in men, who may consciously or unconsciously underreport stress symptoms in self-reports to conform to masculinity norms emphasizing stoicism and strength [173,174].

When interpreting the results of this study, several limitations should be considered. First, the small sample size (due to strict inclusion/exclusion criteria) limited statistical power and may have increased the risk of type II error (failure to detect true effects). Second, the data may be biased by unmeasured confounders. Third, the outcomes are based on clinical pregnancies rather than live births. Overall, this research highlights associations among variables but cannot establish causality.

Further studies with larger sample sizes and well-designed protocols are needed to confirm causal links between male stress and female reproductive function.

## 4. Materials and Methods

### 4.1. Study Design and Participants

The prospective pilot study included twenty-four couples with tubal infertility who underwent ICSI treatment between February 2019 and November 2020. The inclusion criteria for female patients were age ≤ 37 years at the cycle initiation, tubal factor infertility, 0–2 previous IVF attempts, and normal ovarian reserve testing by evaluation of serum levels of AMH and FSH. The inclusion criteria for men were age ≤ 48 years, no extragenital pathology, no severe male factor infertility, including azoospermia or severe oligoasthenoteratozoospermia (<10 million total motile sperm count or <2% strict normal morphology), and no antisperm antibodies.

The female patients were not enrolled in the presence of any of the following exclusion criteria: clinical signs of any autoimmune disease, uterine abnormalities (e.g., adenomyosis, submucous myoma, septate uterus, and endometrial polyps), and unoperated hydrosalpinx.

The exclusion criteria for women were also TORCH (TOxoplasmosis, Rubella, Cytomegalovirus and Herpes) infections and sexually transmitted diseases with Mycoplasma genitalium, Chlamydia trachomatis, Neisseria gonorrhoeae, Trichomonas vaginalis, Treponema pallidum, human immunodeficiency virus, and hepatitis B and hepatitis C viruses.

Male patients with clinical signs of genital tract inflammation, varicocele, leukocytospermia, or any autoimmune disease were excluded from the study.

Additional exclusion criteria for both male and female patients included in the present study were preexisting or current medical or psychiatric conditions, body mass index (BMI) > 30 and <18 kg/m^2^, current medications, smoking, and regular high alcohol consumption.

During the controlled ovarian stimulation phase, patients were advised to engage in 2–3 instances of sexual intercourse, with sexual activity discontinued 3–4 days prior to the scheduled Day-OPU. Compliance with this recommendation was confirmed by questionnaire.

Basal hormone levels, such as FSH, LH, and AMH, were determined on day 2 or 3 of the menstrual cycle preceding the ICSI cycle.

### 4.2. Controlled Ovarian Stimulation

The study group was treated according to a multidose GnRH antagonist protocol. All patients received daily injections of 150–300 IU of rFSH (Gonal-F, Serono Laboratories, Aubonne, Switzerland). The rFSH dosage was adjusted according to follicular growth. A daily 0.25 mg dose of the GnRH antagonist cetrorelix (Cetrotide, Serono Laboratories, Idrone, France) was administered when at least three follicles reached 14 mm and continued until the day of the ovulation trigger. Ovulation was triggered using 10,000 IU of human chorionic gonadotropin (hCG) (Pregnyl, N. V. Organon, Oss, The Netherlands) when at least three follicles reached 17–18 mm, as detected using ultrasound. EMT was assessed on the day of ovulation trigger administration using a transvaginal ultrasound in a sagittal section perpendicular to the midline between the endometrial and myometrium layers on the day of ovulation triggering. All transvaginal ultrasound examinations were performed using a BK Medical Flex Focus 500 ultrasound device (Herlev, Denmark).

Transvaginal ovarian puncture (TVP) was performed 35–36 h later. Immediately after TVP, fresh partner’s SP (0.5 mL) was injected into the posterior vaginal fornix of the patients.

Collected oocytes were inseminated via conventional ICSI. The total number of oocytes retrieved on the Day-OPU, the number of mature oocytes indicated by the presence of a polar body, and the number of normally fertilized oocytes were recorded. Fertilized oocytes were identified by the presence of two pronuclei during fertilization checks, typically observed 16–20 h after insemination.

Blastocyst quality was assessed based on the criteria advocated by Gardner and Schoolcraft [175]. All embryo transfers (ETs) were single ET and occurred 5 days after oocyte retrieval and were performed under ultrasound guidance. All patients received progesterone support in the luteal phase.

Pregnancy was detected using a serum hCG test on day 14 after ET. Clinical pregnancy was defined as a visible intrauterine pregnancy sac examined using ultrasound at 5–6 weeks’ gestational age.

### 4.3. Specimen Preparation and Evaluation

Biofluid samples were collected from male ICSI patients on the Day-OPU. Blood, saliva and semen samples were collected sequentially at approximately 15-min intervals between 10:00 and 11:00 a.m. Unstimulated whole saliva was collected by passive drool into high-quality polypropylene vials. Saliva samples were aliquoted and stored at −80 °C until use in assays.

Fresh semen samples were collected by masturbation into sterile containers after 3–4 days’ abstinence and were analyzed immediately after liquefaction, according to the World Health Organization (WHO) protocol [176]. Conventional semen analysis was performed. Spermatozoa motility was evaluated using the Makler Counting Chamber. DNA flow cytometry was used to distinguish white blood cells from immature sperm [177]. Patients with leukocytospermia, defined as the presence of 1 × 10^6^ white blood cells/mL [176], were excluded. The direct mixed agglutination reaction (MAR) test [178] was performed using the SpermMar Test (FertiPro, Beernem, Belgium). Only specimens with a MAR percentage ≤ 10% were used. One milliliter of each semen sample was used for separation of the SP from the cell fraction by centrifugation, and the remaining semen was used to prepare spermatozoa for ICSI by density gradient centrifugation [176]. Half a milliliter of SP was used for intravaginal administration. All SP samples were then aliquoted and stored at −80 °C until experimental analysis.

### 4.4. Measurement of Catecholamines

A commercially available enzyme immunoassay was used to measure seminal DOP, NA, and ADR levels using the Alpco Diagnostics (Salem, NH, USA) Tri-Cat assay (Cat. No. 17-TCTHU-E03.1). The limits of detection (LOD) were 49 pg/mL for DOP, 36 pg/mL for NA, and 10 pg/mL for ADR. The concentration of DOP was below the LOD in two samples of SP and was replaced with a value of LOD divided by a square root of 2.

### 4.5. Measurement of Cortisol

Cortisol enzyme-linked immunosorbent assay (ELISA) kits were supplied by DBC Diagnostic Biochem (London, ON, Canada). The instruction manuals that were included with the kits for the procedures were strictly followed. CORT levels were measured using a cortisol ELISA kit for saliva (Cat. No. CAN-C-290, the assay sensitivity was equal to 1.0 ng/mL). A cortisol ELISA kit for blood serum (Cat. No. CAN-C-270, assay sensitivity was equal to 4.0 ng/mL) was used for blood serum and SP.

### 4.6. Measurement of Interleukin-18

IL-18 levels were measured by microbead-based flow cytometry assays using a commercially available LEGENDplex Human Cytokine Panel 2 kit (BioLegend, San Diego, CA, USA) and a FACSCalibur flow cytometer (Becton Dickinson, San Jose, CA, USA). Standard curves were generated using the reference cytokine concentrations supplied by the manufacturer. Concentrations of IL-18 (pg/mL) were obtained by interpolating the fluorescence intensities to the standard curves and calculating the concentrations using LEGENDplex™ Data Analysis Software Version 8. The lower LOD of IL-18 detection was 0.7 pg/mL.

### 4.7. Statistical Analysis

Statistical analysis was performed using MedCalc 20.104 and the R statistical environment (version 4.4.0; R Foundation for Statistical Computing, Vienna, Austria) within RStudio (version 2024.12.0.467; Posit Software, PBC, Boston, MA, USA).

Sample size calculations were based on our previous study, which showed high statistical power for associations of stress biomarkers with pregnancy rate in women exposed to their partner’s SP in the IVF/ICSI cycle [21]. With a type I error α = 0.05 and a power of 0.80, we estimated that at least 20 couples undergoing ICSI treatment would be required. Sample size was calculated using MedCalc 22.007.

Multivariate stratification of participants was performed using k-means clustering. Prior to clustering, all variables were standardized using z-score normalization. The optimal number of clusters (k = 2) was determined by minimizing the within-cluster sum of squares and validated via silhouette analysis. To assess k-means clustering robustness with limited sample size, leave-one-out (LOO) sensitivity analysis was performed. For each iteration, one observation was removed, clustering was re-estimated, and partitions were compared to baseline using the adjusted Rand index (ARI). Cluster centroid stability was also evaluated by mean displacement in standardized feature space. Cluster centroids were calculated in the standardized space and subsequently back-transformed into the original units of measurement. The resulting clusters were visualized in three dimensions using the rgl package (v1.3.14).

The normality of the distribution was tested using the Shapiro–Wilk test. The Mann–Whitney test was used to compare continuous variables between the successful and unsuccessful ICSI groups. The Wilcoxon test was applied to compare paired samples within the successful and unsuccessful ICSI groups. Data are presented as the median and interquartile range (IQR). Categorical variables are presented as counts and percentages. Spearman rank-order correlation was applied to calculate the correlation coefficients. Differences were considered statistically significant at *p* < 0.05.

## 5. Conclusions

This study represents an important step toward establishing SP as a unique bodily fluid that integrates male NEI stress signals into female reproductive physiology. NEI stress phenotypes in men reveal a clear dichotomy before and during sexual activity in ICSI patients, manifesting as distinct SP compositions that correlate with reproductive success or failure in women exposed to their partner’s SP during the ICSI cycle. SP likely serves as a biological conduit, transmitting paternal stress phenotypes to the FRT and affecting female reproductive function. Future studies with larger cohorts and refined protocols are needed to confirm these causal pathways, particularly how male seminal stress biomarkers influence key female fertility determinants like folliculogenesis and endometrial growth and receptivity in both ART cycles and natural conception.

## Figures and Tables

**Table 1:** Demographic, clinical, and reproductive characteristics of ICSI patients.

Characteristics	Successful ICSI (*n* = 13)	ICSI Failure (*n* = 11)	*p*-Value
Demographic and clinical characteristics			
Age, male (years)	32.0 (29.8; 33.5)	35.0 (31.3; 41.0)	0.124
Age, female (years)	30.0 (29.0; 34.0)	32.0 (30.0; 34.5)	0.559
BMI, male (kg/m^2^)	25.0 (24.0; 28.0)	26.0 (24.3; 28.8)	0.539
BMI, female (kg/m^2^)	23.0 (20.0; 24.5)	24.0 (20.3; 25.8)	0.598
Duration of infertility (years)	5.0 (3.0; 6.3)	7.0 (5.3; 7.0)	0.132
Basal FSH levels (IU/L)	7.5 (6.6; 9.1)	7.8 (5.9; 9.5)	0.839
Basal LH levels (IU/L)	5.3 (4.3; 7.0)	3.5 (3.1; 5.7)	0.173
Ratio of FSH/LH	1.5 (0.9; 1.7)	1.8 (1.5; 2.0)	0.099
AMH (ng/mL)	2.4 (1.4; 3.3)	2.0 (1.5; 3.0)	0.794
Semen parameters			
Volume, mL	3.5 (2.7; 4.3)	3.3 (2.9; 4.5)	0.977
Sperm concentration (×10^6^/mL)	48.0 (24.8; 90.0)	99.0 (49.0; 111.0)	0.271
Total sperm number (×10^6^)	175.0 (108.3; 250.0)	228.0 (157.3; 378.7)	0.284
Progressive motility (%)	56.0 (49.0; 64.8)	60.0 (48.0; 64.8)	0.772
Sperm morphology (normal forms, %)	3.0 (2.0; 3.0)	2.0 (2.0; 2.8)	0.198
Leukocyte (×10^6^/mL)	0.4 (0.2; 0.7)	0.8 (0.2; 1.3)	0.180
Female fertility characteristics			
EMT (mm)	10.1 (9.3; 10.6)	9.2 (8.6; 10.8)	0.310
No. of oocytes collected	9.0 (7.0; 12.5)	7.0 (6.0; 9.8)	0.179
No. of mature oocytes	8.0 (6.0; 10.3)	6.0 (3.3; 7.8)	0.078
No. of fertilized oocytes	6.0 (5.8; 8.3)	4.0 (3.0; 7.3)	0.091
Male stress biomarkers			
Salivary CORT (ng/mL)	32.8 (28.5; 50.9)	23.3 (19.2; 30.4)	0.012 *
Serum CORT (ng/mL)	83.4 (66.6; 114.6)	52.8 (42.0; 82.0)	0.013 *
Seminal CORT (ng/mL)	58.1 (44.0; 70.6)	45.2 (29.4; 64.4)	0.246
Seminal CORT (total content, ng)	185.8 (161.1; 290.6)	208.0 (88.5; 234.0)	0.531
Seminal NA (ng/mL)	13.8 (5.0; 25.9)	33.0 (18.9; 38.8)	0.077
Seminal NA (total content, ng)	48.5 (20.5; 89.8)	90.0 (59.7; 147.3)	0.087
Seminal IL-18 (pg/mL)	7.8 (4.5; 14.4)	11.9 (9.6; 16.6)	0.247
Seminal IL-18 (total content, pg)	28.1 (24.0; 43.9)	42.8 (28.7; 50.9)	0.247

ICSI: intracytoplasmic sperm injection; BMI: body mass index; FSH: follicle-stimulating hormone; LH: luteinizing hormone; AMH: anti-Müllerian hormone; EMT: endometrial thickness; CORT: cortisol; NA: noradrenaline; IL-18: interleukin-18. * *p* < 0.05.

**Table 2:** Cluster-specific expression of stress biomarkers in male ICSI patients.

Male Stress Biomarkers	Cluster 1 (n = 13)	Cluster 2 (n = 11)	*p*-Value
Salivary CORT (ng/mL)	33.8 (29.5; 51.6)	25.2 (21.2; 28.5)	0.012 *
Serum CORT (ng/mL)	79.6 (58.3; 114.6)	63.4 (42.5; 84.1)	0.264
Seminal CORT (ng/mL)	53.4 (40.2; 70.6)	51.1 (40.0; 65.5)	0.602
Seminal CORT (total content, ng)	187.4 (134.3; 290.6)	178.9 (122.8; 215.0)	0.569
Seminal NA (ng/mL)	9.0 (5.0; 19.0)	35.6 (26.8; 44.1)	<0.001 ***
Seminal NA (total content, ng)	29.4 (18.3; 49.9)	108.8 (80.0; 161.8)	<0.001 ***
Seminal IL-18 (pg/mL)	7.8 (4.5; 13.0)	12.3 (10.0; 26.1)	0.068
Seminal IL-18 (total content, pg)	28.1 (24.0; 37.6)	45.9 (28.8; 80.0)	0.052

ICSI: intracytoplasmic sperm injection; CORT: cortisol; NA: noradrenaline; IL-18: interleukin-18. * *p* < 0.05; *** *p* < 0.0001.

**Table 3:** Demographic, clinical, and reproductive characteristics of ICSI patients by male NEI phenotype.

Characteristics	NEI Phenotype-1 (n = 13)	NEI Phenotype-2 (n = 11)	*p*-Value
Age, male (years)	31.0 (28.8; 33.0)	36.0 (32.3; 43.3)	0.010 *
Age, female (years)	30.0 (28.8; 32.3)	33.0 (30.3; 36.0)	0.031 *
BMI, male (kg/m^2^)	26.0 (24.0; 28.3)	25.0 (24.3; 28.0)	0.838
BMI, female (kg/m^2^)	23.0 (20.0; 26.5)	23.0 (20.0; 24.8)	0.639
Duration of infertility (years)	5.0 (3.0; 6.3)	7.0 (5.3; 7.0)	0.048 *
Basal FSH levels (IU/L)	6.7 (6.4; 9.1)	7.9 (6.2; 9.5)	0.622
Basal LH levels (IU/L)	5.5 (4.3; 7.9)	3.5 (3.0; 5.4)	0.035 *
Ratio of FSH/LH	1.4 (0.8; 1.6)	1.8 (1.5; 2.4)	0.010 *
AMH (ng/mL)	2.7 (1.6; 3.9)	1.7 (1.1; 2.7)	0.099
EMT (mm)	10.2 (9.5; 10.9)	9.2 (8.6; 10.1)	0.042 *
No. of oocytes collected	10.0 (7.8; 14.0)	6.0 (6.0; 7.0)	0.010 *
No. of mature oocytes	8.0 (7.0; 10.3)	6.0 (3.3; 6.0)	0.015 *
No. fertilized of oocytes	7.0 (5.8; 8.3)	4.0 (3.0; 5.8)	0.048 *
Pregnancy rate (n, %)	11 (84.6%)	2 (18.2%)	0.003 **

ICSI: intracytoplasmic sperm injection; NEI: neuroendocrine-immune; BMI: body mass index; FSH: follicle-stimulating hormone; LH: luteinizing hormone; AMH: anti-Müllerian hormone; EMT: endometrial thickness. * *p* < 0.05; ** *p* < 0.01.

**Table 4:** Correlations between biomarkers of male stress and demographic, clinical, and reproductive characteristics of ICSI patients.

Characteristics	Salivary CORT		Seminal NA		Seminal IL-18	
	*r_s_*	*p* Value	*r_s_*	*p* Value	*r_s_*	*p* Value
Age, male	−0.485	0.016 *	0.537	0.007 **	0.441	0.031 *
Age, female	−0.374	0.072	0.421	0.040 *	0.371	0.074
BMI, male	−0.063	0.771	0.088	0.683	0.304	0.149
BMI, female	−0.027	0.901	−0.022	0.919	−0.092	0.668
Duration of infertility	−0.349	0.094	0.506	0.012 *	0.242	0.254
Basal FSH levels	0.122	0.571	0.272	0.198	−0.120	0.576
Basal LH levels	0.203	0.342	−0.358	0.086	−0.039	0.858
Ratio of FSH/LH	−0.015	0.945	0.538	0.007 **	0.116	0.590
AMH	−0.250	0.238	−0.284	0.178	−0.228	0.283
EMT	0.296	0.160	−0.396	0.055	−0.240	0.259
No. of oocytes collected	0.181	0.397	−0.622	0.001 **	−0.209	0.326
No. of mature oocytes	0.265	0.211	−0.533	0.007 **	−0.165	0.442
No. fertilized of oocytes	0.275	0.194	−0.414	0.044 *	−0.015	0.944

ICSI: intracytoplasmic sperm injection; CORT: cortisol; NA: noradrenaline; IL-18: interleukin-18; BMI: body mass index; FSH: follicle-stimulating hormone; LH: luteinizing hormone; AMH: anti-Müllerian hormone; EMT: endometrial thickness. *r_s_*—Spearman’s rank correlation coefficient. * *p* < 0.05; ** *p* < 0.01.

## Data Availability

The original contributions presented in this study are included in the article. Further inquiries can be directed to the corresponding authors.

## Abbreviations

The following abbreviations are used in this manuscript:

ADR	Adrenaline
AMH	Anti-Müllerian hormone
ARs	Adrenoreceptors
ART	Artificial reproductive technologies
BMI	Body mass index
CORT	Cortisol
Day-OPU	Day of ovum pickup
DOP	Dopamine
ELISA	Enzyme-linked immunosorbent assay
EMT	Endometrial thickness
ET	Embryo transfer
FRT	Female reproductive tract
FSH	Follicle-stimulating hormone
GnRH	Gonadotropin-releasing hormone
hCG	Human chorionic gonadotropin
HPA	Hypothalamic–pituitary–adrenal axis
HPG	Hypothalamic-pituitary-gonadal axis
ICSI	Intracytoplasmic sperm injection
IL-18	Interleukin-18
IQR	Interquartile range
IVF	In vitro fertilization
LH	Luteinizing hormone
LOD	Limits of detection
MAR	Mixed agglutination reaction
NA	Noradrenaline
NEI	Neuroendocrine-immune system
PCOS	Polycystic ovary syndrome
SNS	Sympathetic nervous system
SP	Seminal plasma
TVP	Transvaginal ovarian puncture
WHO	World Health Organization
